# Current approaches to characterize micro- and macroscale circuit mechanisms of Parkinson’s disease in rodent models

**DOI:** 10.1016/j.expneurol.2022.114008

**Published:** 2022-02-09

**Authors:** Yangfan Peng, Nina Schöneberg, Maria Soledad Esposito, Jörg R.P. Geiger, Andrew Sharott, Philip Tovote

**Affiliations:** a Institute of Neurophysiology, Charité – Universitatsmedizin Berlin, Charitéplaiz 1, 10117 Berlin, Germany; b Department of Neurology, Charité – Universitatsmedizin Berlin, Charitéplaiz 1, 10117 Berlin, Germany; c Medical Research Council Brain Network Dynamics Unit, Nuffield Department of Clinical Neurosciences, University of Oxford, Mansfield Road, Oxford OX13TH, United Kingdom; d Institute of Clinical Neurobiology, University Hospital Würzburg, Versbacher Str. 5, 97078 Würzburg, Germany; e Medical Physics Department, Centro Atomico Bariloche, Comision Nacional de Energia Atomica (CNEA), Consejo Nacional de Investigaciones Cientificas y Tecnicas (CONICET), Av. E. Bustillo 9500, R8402AGP San Carlos de Bariloche, Rio Negro, Argentina; f Center for Mental Health, University of Würzburg, Margarete-Hoppel-Platz 1, 97080 Würzburg, Germany

**Keywords:** Circuitopathy, DBS, Motor, Circuit, Brainstem, Basal ganglia, Patch-clamp, Silicon probe, Calcium imaging, Optogenetics

## Abstract

Accelerating technological progress in experimental neuroscience is increasing the scale as well as specificity of both observational and perturbational approaches to study circuit physiology. While these techniques have also been used to study disease mechanisms, a wider adoption of these approaches in the field of experimental neurology would greatly facilitate our understanding of neurological dysfunctions and their potential treatments at cellular and circuit level. In this review, we will introduce classic and novel methods ranging from single-cell electrophysiological recordings to state-of-the-art calcium imaging and cell-type specific optogenetic or chemogenetic stimulation. We will focus on their application in rodent models of Parkinson’s disease while also presenting their use in the context of motor control and basal ganglia function. By highlighting the scope and limitations of each method, we will discuss how they can be used to study pathophysiological mechanisms at local and global circuit levels and how novel frameworks can help to bridge these scales.

## 1 Introduction

### 1.1 Parkinson’s disease is a brain network disease that results in diverse motor and non-motor symptoms

Both aggregation of alpha-synuclein and loss of dopaminergic neurons within the substantia nigra pars compacta (SNc) represent hallmark pathologic processes of Parkinson’s disease (PD), which lead to a diverse and progressive clinical phenotype (Kalia and Lang, 2015). This primary pathology results in bradykinesia and rigidity, the cardinal symptoms of PD that are most closely associated with dopaminergic deficit and pathological activity in the basal ganglia (BG) (Kühn et al., 2004; Little and Brown, 2014). Recently, however, circuit-centered research has revealed wider network dysfunctions in PD by highlighting the importance of cell-type heterogeneity, synaptic plasticity and BG interactions with other subcortical nuclei in PD pathophysiology (McGregor and Nelson, 2019; Wichmann, 2019). In addition, it is long known that next to SNc dopaminergic (DA) neurons, other monoaminergic brain regions are affected in the course of PD. While electrophysiological and functional MRI recordings from patients provide valuable disease-specific information, the techniques available in experimental animals allow investigation of the cell-type specific mechanisms underlying PD symptomatology. Taken together, modern circuit neuroscience approaches strongly support characterization of PD as a circuitopathy, i.e. a disease that arises from dysfunction of a brain-wide network of circuit elements (McGregor and Nelson, 2019). In essence, it emphasizes that dysfunction within individual circuit elements causes changes within the entire network, thereby inevitably affecting multiple functions. Furthermore, this perspective helps to understand insensitivity of some PD symptoms to DA replacement therapy and its unwanted side effects, possibly due to plastic changes within the network. Thus, it bears great promise for refinement of existing and development of novel treatment strategies.

### 1.2 Rodent models mimic different pathophysiological aspects of PD

In the following sections, we will focus on the use of rodent animal models due to their wide adoption in the field and the availability of an extensive repertoire of genetic tools to investigate circuit (dys)function in PD. Neurotoxins, such as 6-hydroxydopamine (6-OHDA) and 1-methyl-4-phenyl1,2,3,6-tetrahydropyridine (MPTP), induce a selective degeneration of dopaminergic neurons in the SNc of rats and mice leading to motor impairment (El-Gamal et al., 2021). They are ideally suited to study dopamine replacement and symptomatic treatments of PD, such as deep brain stimulation (DBS), but can not mimic the progressive disease development or non-motor symptoms. Differently, the MitoPark mouse model, that is based on inactivation of a specific pathway in dopaminergic neurons, exhibits progressive motor symptoms which are also responsive to dopaminergic therapy (Ekstrand and Galter, 2009). Another approach is to model PD alpha-synuclein pathology through the use of transgenic or viral vector-based rodent models or by inoculation of alpha-synuclein preformed fibrils. Moreover, several transgenic mice carrying mutated gene variants associated with PD have been developed (Dawson et al., 2018). Genetically based models develop many clinical and biochemical features of PD, but often fail to reproduce motor impairment or a loss of dopaminergic neurons (Koprich et al., 2017). While different models have specific advantages and limitations (for detailed review, see Dawson et al., 2018 and Koprich et al., 2017), the availability of more precise experimental approaches in rodents led to the establishment of key pathological changes in neuronal activity of the basal ganglia network and beyond (McGregor and Nelson, 2019).

### 1.3 Pathological neuronal activity is shaped by the interaction between local microcircuits and long-range pathways

Anatomical tracing methods have established an extensive network of brain regions involved in motor control, including and extending beyond the interconnected basal ganglia nuclei. For example, the striatal direct, indirect and hyperdirect pathways have been identified to serve distinct but complementary functional roles in movement (Cui et al., 2013). Novel approaches to specifically perturb these pathways have greatly expanded our ability to study their macroscale circuit functions (Gittis and Yttri, 2018). On the cellular level, these pathways provide synaptic input to individual neurons of target regions with specific local synaptic connectivity rules that are often not well characterized. While previously these levels were often studied in isolation, a combination of novel methods, such as cell-type specific perturbation with highly resolved single-cell recordings, can be leveraged to gain a more holistic understanding of their function and interaction. We will describe classic and novel methods available to record and perturb neural activity at the cellular microcircuit and global circuit level. In doing so, we will showcase exemplary studies that applied those methods to study motor systems and PD pathophysiology.

### 1.4 The emerging network perspective is key to refining network modulating therapies such as DBS and to identify novel target regions

DBS is a neurosurgical treatment using chronically implanted electrodes to deliver therapeutic electrical stimulation to specific brain regions, such as the subthalamic nucleus (STN), the internal globus pallidus (GPi) or the ventral intermediate nucleus (VIM) of the thalamus. It has been successful in treating motor symptoms of PD, dystonia and essential tremor with current efforts expanding the indications beyond movement disorders (Lozano et al., 2019). Studies on the effects of DBS have greatly contributed to the concept that a localized intervention has a profound impact on brain-wide networks. More specifically, DBS has the potential to bias some aspects of the network towards the healthy state or create novel states that result in an alteration of disease symptoms, as occurs in the parkinsonian brain following dopamine replacement (Heimer et al., 2006). The proposed mechanisms of DBS range from local changes in single cell firing, alterations of synaptic plasticity, to modulation of pathological oscillatory activity on the network level (Ashkan et al., 2017). The complex interaction of DBS mechanisms between the micro- and macroscale and its potential clinical use in a range of neuropsychiatric disorders makes this therapy particularly interesting for circuit neuroscience and its techniques (Gittis and Yttri, 2018; Trevathan et al., 2020a, 2020b). In this review, we will highlight studies that use electrophysiological and optogenetic methods to study DBS mechanisms and how these approaches could be used to optimize current DBS therapies and open new avenues of DBS for treatment of other brain diseases (Creed et al., 2015).

## 2 Neural recording techniques

### 2.1 Ex vivo electrophysiology

**Fig. 1:**
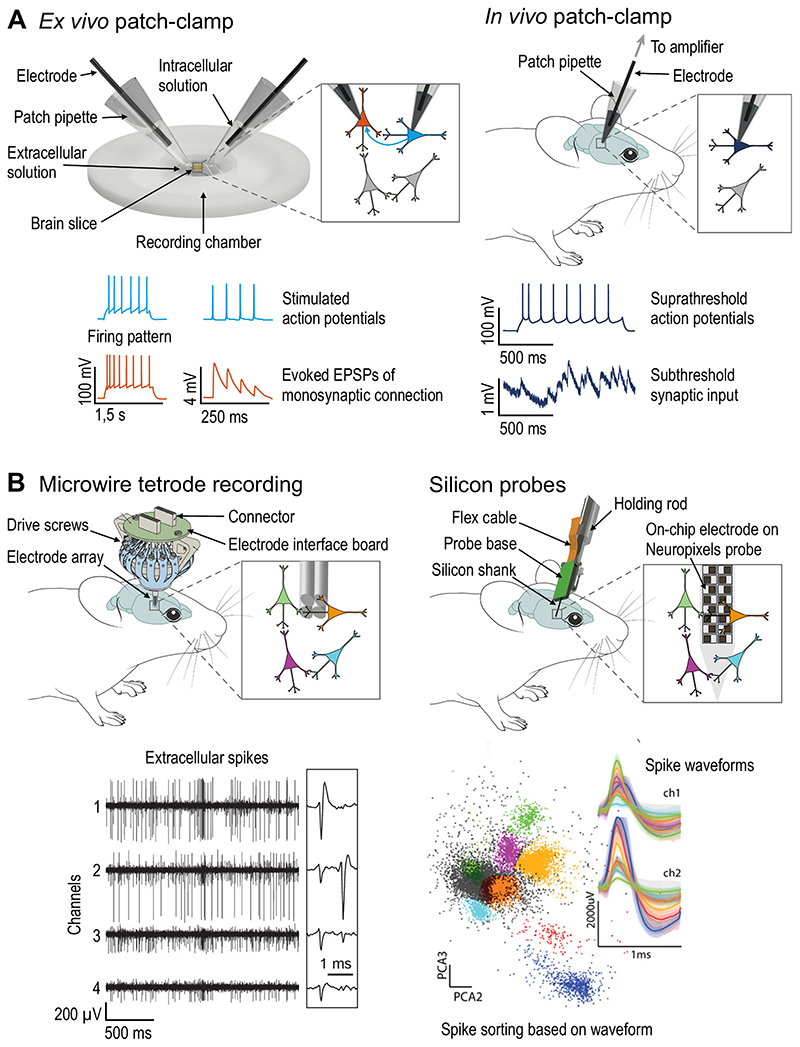
Electrophysiological methods for single cell and population recording. (A) Schematic of patch-clamp techniques and example recordings: *Ex vivo* patch-clamp allows stable recordings from multiple neurons to characterize intrinsic and synaptic properties. *In vivo* patch-clamp allows recording of action potential and synaptic inputs from post-hoc identified neurons in awake animals. (B) Schematic of *in vivo* extracellular recordings and example recordings: Chronic extracellular recordings with microwires allows recording of single unit activity during behaviour. Dense electrode spacing in silicon probes (here Neuropixels probe depicted) allows recording of spikes from many units. For further details on methods, see Table 1. Artwork adapted from Dhawale et al., 2017; Juavinett et al., 2019; Peng et al., 2019; Voigts et al., 2013 under the Creative Commons Attribution License (CC BY 4.0).

A classic approach to study neuronal activity is based on the recording of electrical signals. When the recording electrode is in contact with the intracellular space, small amplitude subthreshold synaptic currents/potentials and suprathreshold action potentials (AP) can be measured, whereas extracellular recordings can only detect neuronal spikes (Fig. 1). In the following sections we will summarise the power and limitations of different electrophysiological methods used to assess single neuron and circuit function.

*Ex vivo* patch-clamp recording provides detailed information about electrical neuronal activity of single neurons. This technique allows direct recording of fluctuations in membrane potential or current at a high temporal resolution which enables for unambiguous detection of ion channel conductances, single APs and subthreshold excitatory or inhibitory postsynaptic potentials (EPSP/IPSP, Fig. 1A, Table 1). Subsequent staining for cells filled with biocytin further allows detailed morphological analyses of the recorded neurons. Typically, patch-clamp recordings are performed on acute *ex vivo* brain slices from rodents that are kept alive by continuous perfusion with oxygenated artificial cerebrospinal fluid. This allows targeting of visualized single cells and controlled application of pharmacological agents to assess the identity of synaptic receptors or ion channels. However, cutting the brain into slices severs afferent and efferent axons restricting the focus of classic patchclamp studies to local cellular and synaptic mechanisms.

Pharmacological interventions during patch-clamp recordings help identify molecular mechanisms underlying specific spiking patterns in the basal ganglia. Patch-clamp recordings found that in the subthalamic nucleus (STN), the transition to burst spiking mode was dependent on inactivating sodium channels (Beurrier et al., 2000). As this technique can also record subthreshold membrane potential fluctuations, it was able to capture prolonged depolarizations in STN neurons after AP firing (Kass and Mintz, 2006). Combining patch-clamp recordings with high frequency electrical stimulation (HFS) in the STN could show that it led to long-lasting changes in postsynaptic currents and burst behaviour in the downstream globus pallidus interna (GPi) (Ammari et al., 2011; Luo et al., 2018). In the striatum (STR), patch-clamp recordings combined with the application of dopamine was able to identify the molecular mechanisms underlying physiological and pathological spiking patterns which could be associated to levodopa (L-DOPA) induced dyskinesia (LID) (McKinley et al., 2019; Paz et al., 2021). Beyond active spiking properties, patch-clamp recordings also allow extensive characterization of intrinsic electrophysiological properties together with detailed morphological reconstruction of neurons in PD models (Branch et al., 2016; Choi et al., 2020; Tubert et al., 2016). For example, striatal projection neurons (SPN) in PD have been shown to exhibit increased excitability and concurrent changes in their morphology, such as reduction of dendritic arborization and spine density (Fieblinger et al., 2014; Suárez et al., 2014).

Multi-neuron patch-clamp recording reveals the synaptic properties of local microcircuits. To record and characterize functional monosynaptic connections in patch-clamp recordings, it is necessary to elicit action potentials in a presynaptic neuron and simultaneously record subthreshold responses in the postsynaptic neuron. This can be achieved by recording from multiple neurons within the same brain slice (Table 1). Stimulating one neuron while recording postsynaptic responses in neighbouring neurons allows detection of excitatory and inhibitory synaptic connections (Qi et al., 2020). This “multipatch” approach has been successful in characterizing properties of synapses between different local cell types in various brain regions (Jiang et al., 2015; Peng et al., 2017; Peng et al., 2021). As connection probability between neighbouring neurons is rather sparse, increasing the number of simultaneously recorded neurons can greatly increase the number of connections probed at the same time. Recent developments of pipette cleaning for reuse and automation of experimental steps have made this previously laborious and technically challenging method feasible to extensively study the local synaptic connectivity (Kodandaramaiah et al., 2016; Kolb et al., 2016; Koos et al., 2021; Peng et al., 2019). In the STN, this approach has uncovered a lack of local connectivity suggesting STN neurons to represent parallel processing units (Steiner et al., 2019). As other basal ganglia nuclei contain mostly inhibitory neurons, this method has been used to study the role of polysynaptic inhibition in the striatal microcircuit (Dorst et al., 2020). While recordings of SPN pairs have shown altered recurrent connectivity in PD (Taverna et al., 2008), there is a continued need for a more complete assessment of microcircuit changes in PD or induced by DBS.

Combining patch-clamp recordings with electrical or optogenetic stimulation can uncover pathway-specific synaptic properties and pathological plasticity changes. Another way to elicit presynaptic action potentials to study synaptic inputs is by electrical stimulation of afferent axonal tracts. This can simulate the impact of upstream regions even if they are not within the brain slice. Such approaches were combined with pharmacological interventions to identify the contribution of NMDA-receptor subunits and D5-receptors for synaptic transmission in STN neurons (Froux et al., 2018; Swanger et al., 2015). Monitoring the change of synaptic amplitudes after repeated stimulation can further uncover short-term plasticity rules in the STN (Baufreton and Bevan, 2008; Steiner et al., 2019) and changes in corticostriatal plasticity in PD (Picconi et al., 2003). Presynaptic axons can also be excited using optogenetic methods that utilize light-sensitive ion channels, allowing cell type- and pathway-specific excitation of afferent inputs (for details on optogenetic methods, see dedicated sections below). Combining patch-clamp with specific optogenetic stimulation of local subpopulations or long-range projections allows investigation of local network connectivity and its interaction with global circuits. In the STR, this approach was used to study local synaptic connectivity between different types of neurons (Straub et al., 2016) or the organization of long-range afferent projections onto striatal neurons (Johansson and Silberberg, 2020). In PD models, this strategy revealed pathological changes in the thalamostriatal synapse (Tanimura et al., 2019 et al., 2019) and in the synaptic transmission from GPe to STN and SNr neurons (Lilascharoen et al., 2021). Further, combining optogenetic stimulation with virally-mediated overexpression of receptors, a recent study was able to rescue PD-related dysfunction of excitation in striatal cholinergic interneurons (Cai et al., 2021). This highlights the potential of combining the subthreshold resolution of the established patch-clamp technique with novel molecular tools to gain a deeper understanding of synaptic pathophysiology.

### 2.2 In vivo electrophysiology

*In vivo* single cell recordings can identify cell-type specific spiking patterns that arise within an intact or pathologically altered circuit. Brain slices are useful to study local mechanisms in controlled and isolated systems, but *in vivo* recordings are necessary to understand the function of neuronal activity that arises from the complex interaction between local and long-range inputs across dynamical brain states. Sharp electrode and patch-clamp recordings can be used to detect sub-threshold activity while *in vivo* multi-neuron patch-clamp recordings are able to characterize synaptic transmission in an intact brain (Böhm et al., 2015; Jouhanneau et al., 2018; Konopacki et al., 2003). Analogous to the *ex vivo* condition, filling neurons can serve post-hoc identification and morphological characterization. *In vivo* patch-clamp recordings have been used to characterize the subthreshold activity of dopaminergic neurons (Otomo et al., 2020). Combined with optogenetic stimulation of afferent regions, such recordings can determine *in vivo* celltype specific synaptic input patterns from these regions in STR (Filipović et al., 2019) or GPe (Ketzef and Silberberg, 2021). While *in vivo* patch-clamp offers subthreshold resolution, reduced recording stability and duration with increased background synaptic activity makes more complex and long-term experiments technically challenging.

The preferred and more robust method to record spiking activity from identified single neurons *in vivo* is by extracellular recordings using glass pipettes without rupturing the cell membrane (juxtacellular recording). Positioning the electrode juxtacellularly still allows the recorded neuron to be labelled with neurobiotin to pair precise post-mortem genetic, neurochemical or structural characterization with its *in vivo* electrophysiological properties. Such recordings have been used to characterize cell-type specific spiking activity in GPe (Mallet et al., 2012) and STR (Doig et al., 2014; Sharott et al., 2012). In rodent PD models, indirect pathway SPN exhibited pathological synchronization (Sharott et al., 2017) and a hyperresponsiveness to cortical and thalamic stimulation (Escande et al., 2016) while spiking activity in the down-stream entopeduncular nucleus (EPN, mouse analogue to human GPi) were abnormally correlated with STN hyperactivity (Aristieta et al., 2019). In addition, pathological changes in this indirect pathway likely increases the sensitivity of the STN to beta frequency activation (Baaske et al., 2020). Combining optogenetics with juxtacellular recordings in PD rats has been used to identify the GP as a critical hub driving pathological spiking patterns and synchronization within basal ganglia networks (Crompe et al., 2020). Although juxtacellular recording can be performed in head-fixed animals (Dodson et al., 2015; Dodson et al., 2016; Delaville et al., 2015; Mallet et al., 2016), the majority of studies have been performed under anesthesia to increase stability.

Single unit and local field potential recordings (LFP) using extracellular microelectrodes can monitor the local population activity during movement and motor symptoms. Microwire electrodes in the extracellular space can detect large and fast voltage changes generated by nearby APs as spiking activity (Fig. 1C, Table 1). Multiple twisted wires (such as tetrodes) further allow triangulation of these signals. Spikes originated from an individual neuron (single unit) can be assigned based on mathematical algorithms commonly referred to as *spike sorting* (Rossant et al., 2016). At the same time, the recorded LFP reflects the superposition of multiple current sources within the local volume of tissue and is widely used as a proxy for local synaptic drive and population activity (Buzsáki et al., 2012). These recordings can be performed in awake and freely moving animals which allows simultaneous observation of single unit and LFP activity during motor behaviour and deficits over several days. LFP recordings established that dopamine-depleted rats, like human PD patients, exhibit neuronal oscillations in the beta frequency range in the basal ganglia (Avila et al., 2010; Delaville et al., 2015; Mallet et al., 2008). Recordings in PD rats found that only subthalamic but not cortical beta power was strongly correlated with dopaminergic neurodegeneration (Haumesser et al., 2021) and that beta oscillations in STR are enhanced during performance of a learned task which could be normalized by L-DOPA treatment (Lemaire et al., 2012). However, beta oscillations or increased burst spiking has not been observed in awake-behaving mouse models of PD (Lobb et al., 2013; Lobb and Jaeger, 2015). Chronic recordings from motor cortex and STR during a motor learning task found that more single units in both regions became phase-locked to the correlated LFP signal with training (Lemke et al., 2019). While spike timing and rate of single units relative to LFP oscillations during behaviour can inform the functional embedding of single neurons in the local network, the low unit yield prevents studying simultaneous activity of larger neuronal populations.

High-density silicon probes increase the number of recorded neurons allowing investigation of local neuron population activity and the impact of long-range inputs. Advances in microfabrication technology enabled commercial production of probes with tens to hundreds of closely spaced recording sites (Berényi et al., 2014; Buzsáki et al., 2015). These silicon probes allow dense sampling of brain tissue and recording of many more local spiking neurons at the same time. Such recordings established the synchronization of GPe and STN units to exaggerated beta oscillations in anesthetized parkinsonian rats (Mallet et al., 2008). These recordings can be further combined with optogenetic stimulation of distant brain regions to identify the impact of a specific pathway on the activity of a local neuron population. This approach has been used to demonstrate firing rate changes in the motor cortex induced by pathway-specific activation of striatal neurons (Oldenburg and Sabatini, 2015). Silicon probes with integrated LEDs at the site of the electrodes further allow spike recordings of many neurons at the site of local optogenetic stimulation (Kim et al., 2020). This approach can be leveraged to identify the neuronal subtype (Lima et al., 2009; Ryan et al., 2018) or to study *in vivo* monosynaptic transmission (English et al., 2017). Silicone probes are thinner and cause less tissue damage than microwires. Although silicon probes can be used in freely moving animals, their high cost biases use towards head-fixed settings, potentially limiting their applicability for studies of motor symptoms. Nonetheless, the increasing advancement of recording methods requiring head-fixation (also see 2-photon calcium imaging introduced below) has been accompanied by the introduction of sophisticated head-fixed motor tasks (Galiñanes et al., 2018; Belsey et al., 2020; Wagner et al., 2020).

More recently, the integration of active electronic components and a complementary metal-oxide-semiconductor (CMOS) based fabrication process have led to the development of Neuropixels probes with a higher electrode density. The first version has one 10 mm shank with 960 recording sites that can be selected for 384 channels and the newer 2.0 version accommodates 5120 recording sites distributed over four shanks with 384 channels for each shank (Fig. 1D, Table 1, Jun et al., 2017; Steinmetz et al., 2021). This technological advance now allows recording of hundreds of neurons simultaneously which opened up new possibilities to study large scale neuronal populations at millisecond timescale. On the cellular level, simultaneous multi-regional recordings could show that neurons coding for choice, action and also internal states can be distributed across many brain regions (Steinmetz et al., 2019; Allen et al., 2019). The increased sample sizes further allow studying of interactions between neurons at the timescale of spikes and monosynaptic transmission to identify functional connectivity within and across brain regions (Siegle et al., 2021). Another approach is to describe the neuronal population dynamics in a multi-dimensional state space where the activity of each neuron is represented as a vector. This perspective has been useful to understand how thalamic input and local dynamics regulate the activity of motor cortical neurons in generating forelimb kinematics (Sauerbrei et al., 2019). Neuropixels recordings can be further combined with calcium imaging (introduced below) to identify the topography and concurrent activity between the cortex and the STR at high spatio-temporal resolution (Peters et al., 2021).

The advent of electrophysiological recordings from hundreds of neurons *in vivo* at spiking resolution opens up new and exciting ways to study neuronal activity at local and global scales (Engel and Steinmetz, 2019). High density recordings in multiple regions in rodent PD models could elucidate how the functional connectivity between and within motor and non-motor regions are affected under dopamine depletion or during motor symptoms. Studying the high-dimensional neural dynamics could further provide a new perspective on how previously established neural signatures of pathology, such as spike-timing and phase-locking, manifest on the population level. While there have been studies using chronic *Neuropixels* recordings in rodents (Juavinett et al., 2019; Luo et al., 2020), further optimization, such as a dedicated commutators and light-weight solutions for mice, are necessary to make it a feasible approach for the wider community. Finally, continued development of computational techniques are needed to analyse and understand these complex and high-dimensional datasets (Stevenson and Kording, 2011).

## 3 Calcium imaging

**Fig. 2:**
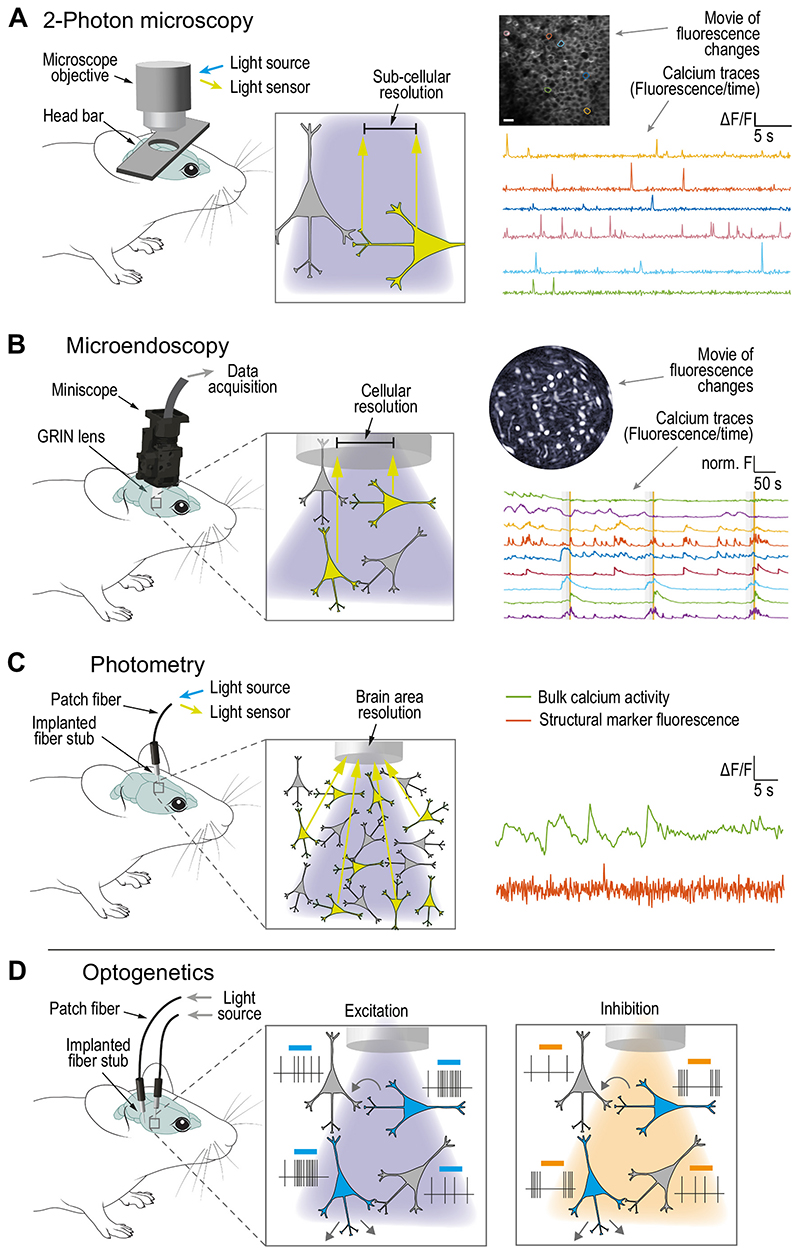
Optical methods for circuit investigations. (A-C) Transgenically or virally-mediated expression of genetically encoded indicators that report intracellular calcium concentration changes by emission of fluorescent light (green arrows) enables recording of neuronal activity in behaving rodents. (A) 2-Photon microscopy allows for imaging of large neuronal ensembles with superior, sub-cellular resolution in head-fixed, behaving animals. (B) Activity of individual neurons within deep regions of the brain in freely moving mice can be imaged by single-photon microendoscopy. (C) Bulk activity of selected neuron populations can be measured via photometry. (D) Optogenetics enables manipulation of selected circuit elements (blue labeled neurons) with high temporal precision. Note that optical manipulation of opsin-expressing neurons will exert local and/or long-range influences onto activity of connected target cells (grey arrows and cells).

Another strategy to assess neuronal activity beyond electrical recordings, is to measure changes in intracellular calcium resulting from APs. Calcium imaging allows cell-specific large scale neural recordings *in vivo*. Genetically encoded calcium indicators (GECI) can report changes in intracellular calcium concentration, termed calcium transients, by enhanced emission of fluorescent light (Table 1–2). By combination of cell-type and/or pathway-specific expression of GECIs with advanced optical methods, neuronal activity can be measured in awake and behaving rodents. Taking advantage of the tight coupling between calcium increase and spiking activity, calcium imaging allows simultaneous recording of the activity of hundreds of neurons simultaneously. This can be done on the level of bulk activity, i.e. combined fluorescent changes of many circuit elements within the targeted brain area, or with single-cell resolution. Below, we will give an overview on three common *in vivo* calcium-imaging approaches and their use in modern basal ganglia research (Fig. 2).

### 3.1 Two-photon imaging

Two-photon imaging allows large scale cell-type specific recording in head-fixed mice. Two-photon microscopy has an outstanding spatial resolution, on the cellular as well as subcellular level, and allows imaging activity of up to 10.000 neurons at the same time (Stringer et al., 2019). However, this imaging approach has most effectively been used only on head-fixed animals as an external objective needs to be placed directly on top of the brain tissue through a craniotomy (Fig. 2A, Table 1). Nonetheless, this approach is compatible with awake and behaving rodents as many tasks have been adapted to the head-fixed setting including motor behavior (also see above section on silicon probes). Few recent studies have reported successful translation of two-photon imaging into freely moving rodents using head-wearable optical devices (Helmchen et al., 2013; Ozbay et al., 2018; Zong et al., 2021; Zong et al., 2017), but further technical developments are needed for broader applicability of this promising technique. By using cell-type specific GECI and considering the greatly increased number of neurons recorded, insights into neural population dynamics are possible (Gritton et al., 2019; Peters et al., 2014; Wagner et al., 2019). Thus, activity sequences time-correlated to specific behavioral patterns were identified within the striatal direct and indirect pathways (Sheng et al., 2019). In PD models, it was possible to show the reduced activity of M1 layer 5 pyramidal neurons in comparison to healthy conditions (Aeed et al., 2021).

In addition to cell-type specific approaches, two-photon imaging is compatible with pathway-specific labeling of cells by conditional expression of GECIs. This allows insights into functional activity of specifically selected circuits. Conditional expression refers to the dependence of expression of genetic material, for example introduced by viral vectors (e.g. AAVs) or carried in transgenic mouse lines, on the presence of specific enzymes. A popular system to be used is the Cre/ loxP system (Table 2), allowing for cell-type and/or projection-specific labeling of neurons. Using this approach, it was shown that the projection of pyramidal neurons from the prefrontal cortex to the STN is involved in the decision-making process of a go no-go motor behavioral assay (Li et al., 2020). Another approach for gaining pathway specificity, is the recording of calcium related cell activity within synapses of targeted cells. In a seminal study, Howe and Dombeck used cell-type specific two-photon calcium imaging to demonstrate functional specificity of dopaminergic neurons for either reward or locomotion (Howe and Dombeck, 2016). In addition, two-photon imaging has been successfully used for chronic calcium imaging allowing the same neurons to be recorded over days (Sheng et al., 2019; Wagner et al., 2019). Presenting a promising experimental avenue, chronic recording in animal models of PD would allow mapping of progressive changes in the disease course on the cellular and circuit level.

### 3.2 Microendoscopy

Microendoscopy reveals structured spatial organization of striatal neurons in freely moving animals. While two-photon imaging provides great spatial resolution, it is limited to superficial brain regions of head-fixed animals. Microendoscopy combines chronically implanted intracranial graded index (GRIN) lenses with miniaturized, head-wearable microscopes (*miniscope*) and allows the observation of calcium-related activity of up to hundreds of single cells in deep brain regions of freely moving animals (Ghosh et al., 2011; Jung et al., 2004; Ziv and Ghosh, 2015). Therefore, it represents a powerful technique for providing insights into movement-encoding neuronal ensembles (Hamel et al., 2015). Using this method, a spatial organization of SPNs correlating with behavioral patterns has been uncovered (Barbera et al., 2016; Klaus et al., 2017). Unilateral dopamine depletion showed increased activity in ipsilateral iSPNs, but reduced locomotion coupling and spatial clustering. These results further illustrate pathologic iSPN activity change following a chronic lack of dopamine (Parker et al., 2018). In addition, the combination of electrical STN stimulation and single photon microendoscopy, neural activity of ipsilateral SPNs in anesthetized and freely moving mice in a PD model could be recorded (Trevathan et al., 2020a, 2020b). Although this is a single study, it demonstrates the feasibility of this approach to better understand the effects of DBS. Future refinement of microendoscopy, such as implementation of wireless technologies (Barbera et al., 2019) or weight reduction of equipment (Groot et al., 2020), will increase the animals’ range of motion and facilitate establishment of semi-natural conditions, thereby expanding the application range. However, the combination of complex surgical, viral vector, hardware as well as sophisticated analytical approaches makes microendoscopy most suitable for mechanistically-oriented, basic research applications without the need for high throughput. In particular, while the relation between single-cell calcium transients and neuronal spiking activity has been well characterized in cortical pyramidal neurons (Vanni and Murphy, 2014), this relation remains insufficiently understood for deeper brain regions and different cell types (Wei et al., 2020). Similarly to other techniques requiring intracranial implantations, the mechanical lesion induced by the lens itself (0.5-1mm) presents a systemic caveat of microendoscopy.

### 3.3 Fiber photometry

Fiber photometry demonstrates synchronous activity of direct and indirect pathway neurons. Technically less demanding than microendoscopy, fiber photometry allows recording of calcium-related activity of neuronal subpopulations in defined (deep) brain regions in freely moving mice (Fig. 2C, Table 1). As opposed to microendoscopy, fiber photometry cannot resolve a single neuron’s activity but reveals the calcium-dependent activity of entire populations of cells, identified by their specific molecular or projections-based signature (Sych et al., 2019). Thus, the use of fiber photometry enables investigation of population activity during specific motor behavioral states in a given brain region, so that motor-encoding population vectors, a signal created by a sum of active neurons, can be examined. For example, the expression of GECIs in d/iSPN revealed simultaneous activation of both subpopulations during motor initiation (Cui et al., 2013). In line, the development of a multi-color, multi-site fiber photometry approach, consisting of two different fluorescent calcium indicators introduced into d/iSPN, enabled demonstration of synchronous activity of both pathways in freely moving mice (Meng et al., 2018). Due to the relative minor invasiveness of thin optical fibers (approx. 200 μm) and the small area of space required on the animal’s skull for fixation, placement of multiple optical fibers and thus, recording of several brain regions at the same time is possible (Sych et al., 2019). Importantly, calcium activity can be recorded both, from neuronal cell bodies as well as from axon terminals within their target regions. Using two-site, cell-type specific photometry imaging on axon terminals, functionally distinct pallidal pathways for specific PD symptoms were recently identified. That is, synaptic input by pallidal parvalbumin-positive (PV) neurons connecting to the STR supports locomotion, whereas a PV-positive pallidal pathway to the thalamic parafascicular nucleus underlies reversal learning (Lilascharoen et al., 2021). Similar to calcium imaging via microendoscopy the relation between calcium transients and spiking activity is not always clear. Furthermore, calcium activity within the neuropil may significantly, and depending on the region of interest, even predominantly contribute to the overall calcium signal recorded by photometry (Keemink et al., 2018). The use of soma-targeted calcium sensors would allow to circumvent this confound and to differentiate between somatic and neuropil signals (Chen et al., 2020; Shemesh et al., 2020).

Overall, calcium imaging methodology yields large scale recording of neuronal activity within selected circuit elements such as cell types and their projections, as well as subcellular compartments such as dendritic spines and axonal boutons. While two-photon and micro-endoscopy approaches can reveal complex heterogeneity of individual cellular activity associated with specific functions or events, fiber photometry allows for understanding of average neuronal activity of a distinct cell type at its soma or synaptic level at a given time. This is especially useful for investigation of long-range projection pathways, such as those originating from SPNs. In contrast to electrophysiological techniques, calcium imaging potentially allows observation of a significantly larger number of identified neurons, so that neural ensemble dynamics related to motor behavior can be studied. In addition, further development of fluorescent sensors will not only enable researchers to improve calcium imaging, but also, for example, to measure release of neuromodulators, such as dopamine or serotonin (Feng et al., 2019; Sun et al., 2020). The development of a new generation of genetically encoded voltage sensors promises to overcome the current limitations in temporal precision (Piatkevich et al., 2019). These new tools will un-doubtedly lead to novel insights into monoamine signaling under normal and pathological conditions, including movement disorders.

## 4 Neural perturbation techniques

### 4.1 Optogenetics

Complementing to the observational electrophysiological and calcium imaging recordings, optogenetics is a state-of-art method that allows targeted perturbation of identified cell groups within a specific (deep) brain region and their projections within a circuit by expression and activation of light-sensitive proteins (Fig. 2D, Table 1–2). Since its inception, a large toolbox of light-sensitive actuators (e.g. transmembrane channels, receptors, ion pumps or second messengers), viral vectors and light delivery systems have been developed that led to the application of optogenetics in freely moving animals, including worms, flies, fish, rodents and non-human primates (Deisseroth, 2015). Phase I/II clinical studies for the use of optogenetics to treat retinal disease are ongoing (Simunovic et al., 2019). Optogenetics, because of its superior circuit element-specificity and temporal precision, has greatly empowered analysis of circuit function, i.e. enabled researchers to draw conclusions about activation and inactivation of neuronal populations and corresponding behavioral output (Tye and Deisseroth, 2012). Therefore, this approach could help to identify functional roles of motor networks and elucidate the mechanism of DBS by mimicking therapeutic effects on PD symptoms through specific modulation of motor pathways.

Optogenetic perturbation highlights the role of basal ganglia pathways for distinct motor behavior. It has been widely applied to different BG pathways which identified their differential contribution to movement control (Bouvier et al., 2015; Capelli et al., 2017; Gradinaru et al., 2009; Kravitz et al., 2010; Roseberry et al., 2016; Ruder et al., 2021; Watson et al., 2021). Initially, direct behavioral effects of the activation of d/iSPN were investigated, which resulted in association of pro-kinetic function with the direct pathway, while motor arrest was attributed to activity of the indirect pathway. Furthermore, freezing in PD mice could be reduced by targeted prolonged stimulation of the D1 neurons in the STR (Kravitz et al., 2010). This relatively coarse pro- and anti-kinetic concept was refined by subsequent more detailed experiments using novel *in vivo* calcium imaging techniques and inhibitory opsins (Cui et al., 2013; Parker et al., 2018; Tecuapetla et al., 2016) that highlighted a rather simultaneous interaction of both pathways for motor initiation while also showing the modulatory effect of direct and indirect pathway neurons regarding latency and duration of movement (Tecuapetla et al., 2016). Further, closed loop optogenetic stimulation protocols with an activation of d/iSPNs during different movement speed resulted in bidirectional changes of velocity (Yttri and Dudman, 2016). Here, application of dopamine antagonists abolished modulatory effects, suggesting a dopamine-dependent plasticity mechanism.

Electrically recorded neurons can be identified through optogenetics *in vivo*. Because of its fast kinetics and drastic impact on neuronal function, i.e. imposition or blockade of spiking activity, optogenetics can help elucidate cellular identity in otherwise “blind” extracellular electrical recordings. These photo-tagging approaches, originally developed with a focus on cortical circuits and interneurons (Lima et al., 2009) have been widely applied and opto-electrodes now became standard tools in modern circuit neuroscience (Lee et al., 2020). Refinement of the technology allows combination with the latest, large scale recording approaches (Kim et al., 2020). Nonetheless, especially when applied in freely moving mice, additional considerations as to choice of opsin kinetics and properties of targeted circuit elements, as well as stimulus design and analysis criteria are required to ensure conclusivity of obtained results and subsequent interpretations.

Optogenetics can help to link pathological circuit mechanisms to motor symptoms. Optogenetic perturbation can be used to model Levodopa (L-Dopa) induced dyskinesia (LID), which is the main therapy-limiting adverse effect and very difficult to treat (Picconi et al., 2018). As discussed regarding the mechanisms of DBS, optogenetic stimulation offers the possibility of identifying individual cell type-specific groups that could be responsible for dyskinetic symptoms. So far, neurons of the nigrostriatal pathway targeted by axonal stimulation (Keifman et al., 2019), as well as the combined optogenetic activation of d/iSTR neurons (Hernández et al., 2017) evoked dyskinesia in PD rodent models even without preexposure to L-DOPA. Optogenetic activations of individual striatal subgroups (Girasole et al., 2018; Ryan et al., 2018) and cholinergic interneurons (Bordia et al., 2016) have shown to be sufficient to produce dyskinesia in dopamine-depleted mice. Additionally, L-DOPA treatment effect on optogenetically evoked behavioral phenotype might provide insight into underlying molecular pathomechanisms (Picconi et al., 2018). For example, an increase of dyskinetic behavior following L-DOPA treatment has been reported, suggesting a direct neuro-modulatory link (Perez et al., 2017; Ryan et al., 2018). These examples demonstrated that optogenetics provides a very precise tool for investigating pathophysiological changes in motor circuits caused by depletion or excess of dopamine.

Functional dissection of afferent and efferent projections of the STN demonstrates their potential contributions to therapeutic DBS. Optogenetics is a powerful tool for studying the mechanisms by which DBS modulates neuronal activity in a cell-type and pathway-specific manner, thereby supporting future refinement of stimulation protocols and electrode placement (Gittis and Yttri, 2018; Lüscher and Pollak, 2016). In line with the hypothesis that STN electrical stimulation produces a lesion-like transient effect (Herrington et al., 2016), the optogenetic inhibition using Halorhodopsin, a light-activated chloride ion pump, introduced into the STN resulted in an improvement of akinesia in the 6-OHDA rat (Yoon et al., 2016). Optogenetic stimulation of Pitx2 expressing STN neurons resulted in a reduction of postsynaptic activity in STN projection targets and increased locomotion (Schweizer et al., 2014). In one of the main output regions of the STN, the motor-related GPe, this need for cell-type specific targeting is further highlighted: while optogenetic stimulation of the whole GPe did not rescue PD motor symptoms, optogenetic activation of GPe PV-positive neurons (Mastro et al., 2017; Pamukcu et al., 2020) and inhibition of GPe Lhx6 neurons (Mastro et al., 2017) revealed a locomotion-promoting effect with a potential long-lasting recovery effect. In contrast, optogenetic stimulation of GPe Npas1 neurons reduced locomotion (Pamukcu et al., 2020). This suggests that a selective manipulation of specific subpopulations might be critical for the efficacy of DBS, such as the role of the GPe in mediating aberrant network activity (Crompe et al., 2020; Spix et al., 2021). While these studies have brought many novel insights, there are important limitations when considering their relevance to therapeutic DBS. While the scale of cell-type specific manipulations in rodents is ideal for the dissection of specific circuit elements, therapeutic DBS stimulates a range of different neuronal elements simultaneously (cell bodies, fibres of passage, surrounding structures). Rodent studies can show that manipulating specific cells or synapses is sufficient to affect behaviour in a similar way to DBS, but this does not necessarily indicate that electrical stimulation works through the same mechanism in the human brain. However, recent studies suggest that moving back and forth between optogenetic and electrical stimulation can yield impactful results (Spix et al., 2021).

Optogenetic dissection of the hyperdirect pathway and its long-range projections highlights its role in therapeutic DBS. Considering the network nature of motor function, and beyond a focus on local STN-DBS induced changes, several studies addressed the role of cortical inputs to the STN, the so-called hyperdirect pathway. An early study from Gradinaru et al. reported, comparing electrical stimulation and cell type-specific optogenetic perturbation of STN neurons in 6-OHDA mice, that neither activation nor inactivation of glutamatergic populations in the STN had a therapeutic effect on motor behavior (Gradinaru et al., 2009). However, activation of glutamatergic efferents from the motor cortex (M1) layer V showed improvement of locomotion (Gradinaru et al., 2009). Furthermore, pathological hyperactivity in M1 was also observed during electrical stimulation of the STN (Valverde et al., 2020).

On a microcircuit level, electric STN-stimulation increased firing rates of optogenetically identified somatostatin (SST) interneurons in the M1. Optogenetically activating those SST neurons reduced PD motor symptoms in 6-OHDA mice (Valverde et al., 2020). These results support the hypothesis that the therapeutic effect of motor-based PD symptoms are attributable to an antidromic activation of the hyperdirect pathway (Gradinaru et al., 2009). In line with this notion, but contrasting other findings (see below), specific stimulation of M1 layer 5 neurons that project to STN improved bradykinesia in 6-OHDA mice (Sanders and Jaeger, 2016). Importantly, the STN projections from the motor cortex emerge from collaterals of L5 corticofugal axons that also project to multiple downstream motor regions, raising the question of their involvement in PD symptom modulation.

In the context of STN stimulation, optogenetics was used to understand the impact of stimulation frequency patterns. To support specific frequency protocols, the employed opsin needs to have suitable temporal kinetics (Table 2). A direct correlation between the optogenetic stimulation frequency and motor behavior was described in the corticothalamic pathway: whereas stimulation frequencies of 4 Hz resulted in physiologic movement, higher frequencies caused severe forelimb impairment (Sauerbrei et al., 2019). Further, the direct impact of opsin kinetics on behavioral output measures is highlighted by the finding that optogenetic HFS of ChR2-expressing neurons in the STN had no therapeutic effect (Gradinaru et al., 2009), whereas activation of the same neurons using Chronos, an opsin with faster kinetics, resulted in PD motor symptom reduction (Yu et al., 2020). In addition, HFS (100-130 Hz) in the STN and the M1-STN pathway resulted in improvement of impaired locomotion, while low frequency stimulation (20 Hz) showed no effect (Gradinaru et al., 2009; Sanders and Jaeger, 2016). In a recent elegant study, cell-type specific optogenetic characterization of differential inputs to the GPe was used to identify optimal electrical stimulation patterns for promoting locomotion in a mouse model of PD (Spix et al., 2021). In sum, previous studies using optogenetics illustrated the importance of cell type and pathway specificity, as well as stimulation frequency in generating desired motor outcomes. Optogenetic techniques will continue to elucidate the putative cellular and circuit mechanisms of DBS, thereby building the base for refinement of the technology in treating motor diseases.

Modern circuit-centered neuroscience has uncovered brainstem circuits for locomotor control. Until recently, most studies on PD circuit mechanisms have focused on the BG network. However, the consequent refinement of optical and genetical methodology, such as miniaturisation of hardware and ever-increasing specificity and temporal precision of observational and perturbational tools, now enables systematic investigation of evolutionarily older subcortical brain regions instrumental for motor control. Specifically, brainstem circuits are crucial network elements for regulation of motor functions, such as locomotion and behavioral arrest (Bouvier et al., 2015; Capelli et al., 2017; Roseberry et al., 2016; Tovote et al., 2016). Important from a clinical perspective, motor arrest is a difficult to treat symptom in PD, with some patients experiencing episodes of acute akinesia, called freezing of gait. In addition, the contribution of dysregulated activity within brainstem circuits to non-motor PD symptoms and co-morbidity, e.g. sleep, pain and affective disorders, is receiving increased attention in basic and clinical research. The zona incerta (ZI), located in close proximity to the STN is connected to several brainstem areas (Kolmac et al., 1998). Due to successes in clinical DBS trials targeting the ZI, its putative role in treatment of PD motor symptoms is debated (Ossowska, 2020). In addition, optogenetic studies showed that the ZI has a modulatory role in locomotion (Hormigo et al., 2020) and that its prefrontal input is involved in cognitive control and action selection (Heikenfeld et al., 2020). In line, specific ZI PV neurons targeting the brainstem peri-aqueductal grey (PAG) were important for modulation of flight and freezing behavior (Chou et al., 2018), behaviors that were also shown to be affected by dopaminergic modulation of PAG circuits via cerebellar inputs (Frontera et al., 2020; Vaaga et al., 2020).

Besides behavioral arrest and freezing, brainstem circuits have long been implicated in the control of active locomotor patterns (Ray et al., 2011). More recently, optogenetic perturbation of striatal projection to the mesencephalic locomotor region (MLR) demonstrated a role in modulation of movement speed (Roseberry et al., 2016). As part of the MLR, optogenetic activation of glutamatergic neurons in the cuneiform and the pedunculopontine nucleus resulted in motor speed modulation and their recruitment was associated with flight and explorative situations, respectively (Caggiano et al., 2018). However, glutamatergic pedunculopontine neurons have been also implicated in the negative control of the motor output, as well as in other forelimb-dependent movements (Dautan et al., 2021; Ferreira-Pinto et al., 2021). Importantly, MLR dysfunction has been linked to increased freezing of gait in human PD patients (Fling et al., 2014; Alibiglou et al., 2016; Tubert et al., 2018). MLR dysfunction in PD could arise from the dysregulation of basal ganglia circuits, but also from neurodegeneration of local cholinergic neurons or other input regions affected by the disease, such as noradrenergic neurons from the locus coeruleus (Hirsch et al., 1987; Iversen et al., 1983). In addition, optogenetic stimulation of the glutamatergic neurons of the medullary lateral paragigantocellular nucleus (LPGi) led to forward movement and speed changes (Capelli et al., 2017), while activity of excitatory reticular formation neurons or LPGi inhibitory neurons revealed a link to immediate motor arrest as well as an effect on motor rhythm (Bouvier et al., 2015; Capelli et al., 2017). Beyond locomotion, specialised brainstem circuits control left-right turning and skilled forelimb movements (Cregg et al., 2020; Esposito et al., 2014, Ruder et al., 2021). State-of-the-art fiber photometry and *in vivo* electrical recordings revealed a role of the lateral rostral medulla in forelimb reaching and handling (Ruder et al., 2021). These studies, applying sophisticated combinations of modern circuit-centered approaches to brainstem circuits highlight their role in motor function and the need to incorporate them into models of PD network dysfunctions.

### 4.2 Chemogenetics

Another novel approach to manipulate neuronal activity of specific circuit elements utilizes genetically engineered ion channels or receptors exclusively activated by inert synthetic ligands which can be administered systemically (Armbruster et al., 2007; Magnus et al., 2011; Vardy et al., 2015). Similar to optogenetics, particular receptors can be introduced into specific neuronal subpopulations and pathways to reversibly activate or inhibit neuronal activity. Moreover, chemogenetic approaches do not require chronic intracranial implantations and can be easily combined with any form of neuronal activity recording in freely moving animals (Table 1). Compared with optogenetics, the time course of drug effect in chemogenetics is slow, ranging from minutes to hours. Especially when employing this method for manipulating behavioral readouts, potential unspecific side effects of ligand metabolites have to be controlled for (MacLaren et al., 2016).

Motor symptoms in PD mouse models have been reverted by chemogenetics. Dopamine depletion in PD affects the normal activity of multiple basal ganglia nuclei. Therefore, chemogenetic manipulation of neuronal activity within specific neuron subpopulations can be used to counterbalance circuit dysfunction. For example, selective chemogenetic activation of dSPN was shown to mimic L-DOPA therapeutic effect in 6-OHDA-lesioned mice, whereas chemogenetic stimulation of iSPN reduced LID severity (Alcacer et al., 2017). Selective enhancement of GPe activity or reduction in basal ganglia output activity by silencing GPi and SNr was shown to significantly improve motor performance in 6-OHDA mice (Assaf and Schiller, 2019). Moreover, chemogenetic activation of striatal cholinergic interneurons potentiates L-DOPA therapeutic effect on locomotor function, reduces falls and improves cued turning performance in PD rats (Aldrin-Kirk et al., 2018; Avila et al., 2020). Finally, maladaptive thalamostriatal plasticity triggered by dopamine depletion can be compensated by pathway-specific chemogenetic inhibition, thereby restoring motor function (Parker et al., 2016).

Beyond chemogenetics, novel genetic tools allow inducible inactivation of endogenous proteins to perturb neuronal function and behaviour. In mice, inducing a knockout of D2-receptors during adulthood has been shown to cause PD-like phenotypes with typical pathological neural activity recorded using silicon probes (Bello et al., 2017). Patch-clamp recordings in mice with cell-type specific knockout of D2-receptors were able to show that parkinsonism caused by antipsychotics are mediated via the striatal cholinergic interneurons (Kharkwal et al., 2016). Finally combining modern neural recording methods with state-of-the-art genetic tools that can efficiently induce cell-type specific inactivation of proteins using CRISPR/Cas9 technology (Hunker et al., 2020) will greatly increase our understanding of neural function on the molecular level and help to identify novel therapeutic targets.

## 5 Discussion

Technological advances in recent years have resulted in a vast array of promising neuroscientific tools that allow recording and perturbation of neural activity in unprecedented detail. This development now allows basic and clinical researchers to adequately address the complexity of the mammalian nervous system as a global network of interconnected microcircuits. By combining correlational and causative approaches, thereby functionally characterizing individual circuit elements and their input-output connectivity, truly mechanistic insights into the underpinnings of normal and pathologic neuronal conditions can be gained. In this review, we have introduced a range of advanced electrophysiological, optical, and genetic approaches (Figs. 1–2) that altogether and in combination serve to provide a multifaceted understanding of PD pathophysiology, DBS mechanisms and motor control on the cellular and circuit level. Nonetheless, each method has specific advantages and limitations for the given research question that need to be considered during the experimental planning, execution, and analysis phase (Table 1).

Observational electrophysiological and optical methods provide different spatial and temporal resolutions. When deciding which method to use for a specific research question, it is vital to consider the spatial and temporal scale of the studied effect. Two-photon calcium imaging provides a very high spatial resolution as it can visualize neuronal activity in individual somata and even in subcellular compartments such as axons and dendrites. Furthermore, thousands of neurons can be recorded simultaneously depending on the regions scanned by the laser. Single-photon microendoscopy has a lower spatial resolution, but is suited to study deeper brain regions (Ghosh et al., 2011). However, the temporal resolution of these approaches is limited by the sampling time needed to detect a signal with the microscope, the number of simultaneously sampled pixels and, more crucially, the kinetic of the calcium indicators (half decay time of jGCaMP7f = 265 ms, Dana et al., 2019). Thus, fluorescent calcium signals are yet unable to resolve single action potentials (~ 1 ms), but rather serve as a proxy for events of neural activation. Recently, first studies using fluorescent voltage sensors have demonstrated proof-of-principle in rodent models and promise higher temporal resolution in future circuit studies (Piatkevich et al., 2019; Tian et al., 2021). On the other hand, electrophysiological amplifiers can detect electrical signals on μs scale (filter frequency up to 20 kHz), exceeding the kinetics of the fastest ion channels and action potentials. This makes electrophysiological recordings especially suited to study spike timing and fast subthreshold kinetics (Geiger and Jonas, 2000). However, this increased temporal resolution comes at the cost of a smaller sample size (but see section on Neuropixels probes above) and additional efforts are required to define the exact position and molecular identity of neurons recorded *in vivo*.

Combining optogenetics with optical or electrophysiological large-scale recordings can help to bridge the macro- and microcircuit level. Previous technical constraints limited recordings to only a few hand-selected cells and theoretical frameworks focused on neurons as single computational units with properties such as firing rates or spiking variability (Parker and Newsome, 1998). With the availability of the above introduced high-throughput methods, novel frameworks now incorporate the high-dimensional neural dynamics in local populations and across brain regions (Urai et al., 2022). While those observational recordings can only describe correlative activity, sophisticated analytical approaches comprising detailed information on neural activity of identified cell ensembles, individual motor functions and internal meta-states can support conclusive interpretations even in the absence of causative experiments. Nonetheless, in providing this direct causal approach, optogenetic or chemogenetic perturbation of selected circuit elements, i.e. neuron subgroups and their projections, greatly facilitates mechanistic dissection of how specific local microcircuits and their long-range connectivity contribute to the activity dynamics within entire networks. Combining highly sensitive opsins with fast kinetics and advanced holographic microscopy allows for semi-naturalistic manipulation of neuronal ensembles mimicking their natural activity patterns (Marshel et al., 2019). Importantly, whereas parallel observational and perturbational approaches provide insights by summing up evidence from many individual experiments, the combination of these methods within the same experiment and/or experimental subject yields superior conclusivity.

Modern circuit neuroscience methods have a great translational value in advancing our understanding of PD as a circuitopathy and to develop novel DBS approaches. Recent studies that have manipulated precise circuit elements highlight the ability of optogenetic studies to uncover novel principles of basal ganglia computation (Yttri and Dudman, 2016) and address fundamental questions about pathophysiological mechanisms in PD. For example, optogenetics combined with electrophysiology could delineate the critical contribution of GP but not STN activity to pathological synchronization in the network, despite them being highly interconnected (Crompe et al., 2020). This finding contrasts with the conclusions drawn from previous studies relying mostly on recording and/or lesion of these populations. Another promising use of optogenetics is to identify novel manipulations that produce effects beyond what is possible with existing methods. Optogenetic stimulation of a specific population of PV-expressing GPe neurons in mice can alleviate parkinsonian behaviours for hours after stimulation has ceased (Mastro et al., 2017) and can even be used to improve electrical stimulation protocols (Spix et al., 2021). Furthermore, optogenetics can help identify novel targets for modulation, such as cortical somatostatin interneurons (Valverde et al., 2020). Such insights could not have been gained from electrical stimulation or lesion studies alone and demonstrate that it is possible to translate insights at the level of specific cell-types to clinically relevant findings. Mechanistic understanding of basal ganglia function and DBS can further be exploited to optimize adaptive and closed-loop DBS approaches (Bouthour et al., 2019; He et al., 2021; Yttri and Dudman, 2018). The introduced methods and their continued development together with more refined animal models (Dawson et al., 2018) will be critical to study the interplay of these scales and systems in PD and other neuropsychiatric diseases.

Key questions remain as to how brainstem nuclei contribute to parkinsonian symptoms. Given that cortico-basal ganglia circuits mediate a large proportion of motor output through these areas, their output will inevitably be indirectly affected by pathological changes in those outputs. Ascending projections from the brainstem back to these input areas may then further contribute to network dysfunction. Importantly however, several areas of the brainstem may also be directly affected by neurodegeneration in PD, and this occurs before extensive dopamine depletion (Braak et al., 2004). Cell-type specific manipulation of brainstem populations in mouse models of degeneration could provide a way of delineating between dopamine-mediated circuit dysfunction and neurodegeneration in these areas for development of parkinsonian symptoms.

However, a challenging aspect of perturbational circuit-centered approaches is the nature of the studied object itself - a network of connected circuit elements that interact to generate specific functions. It is therefore important to cautiously interpret effects due to manipulation of individual circuit elements (Otchy et al., 2015). Performing both, gain- as well as loss-of-function experiments on single circuit elements, gathering evidence from multiple network components, and comparing acute with chronic interventions is key to avoid misinterpretation of neural mechanisms underlying complex output measures, such as motor functions or behavioral states (Pernía-Andrade et al., 2021). Bearing promise for translational relevance, optogenetics could be used to insert ‘normal’ activity into key circuits. However, because of the interconnected nature of the system and possible long-term and compensatory effects, normalization of entire network activity remains a challenge. In line, reduced invasiveness of current circuit approaches are crucial for translation into clinically relevant applications, e.g. to use optogenetics the diagnosis or therapy of diseases (Kushibiki, 2021). Furthermore, the refinement of viral vector gene delivery, the development of ultra-sensitive optical actuators (Chen et al., 2021), upconversion of stimulation light using highly penetrable near-infrared light (Chen et al., 2018) as well as novel methods using electric field interference for targeted manipulation of neuronal activity (Grossman et al., 2017) will broaden the technical foundation for less invasive therapies that allow rational interference with neuronal circuits *in situ*.

Finally, the successful translation from mechanistic insights into PD circuit pathophysiology from rodent models towards human patients requires close collaboration between basic scientists and clinicians. This can be achieved within interdisciplinary research consortia, organised around a focus topic, and following unifying working hypotheses. Beyond bringing together researchers from different backgrounds, they also provide ideal environments for the training of a new generation of clinician and medical scientists, which are capable of integrating complex circuit neuroscience with theoretical knowledge of neurodegenerative disease and practical experience of the clinical routine.

## Figures and Tables

**Table 1:** Characteristics of neuroscience methods

Method	Advantage	Limitation
***Ex vivo* whole-cell patchclamp**. A glass pipette containing an electrode and filled with an intracellular solution is moved onto the membrane of a neuron under visual guidance using a micromanipulator. The neuronal membrane at the pipette tip opening is ruptured while the pipette edge forms a seal with the intact membrane. This technique allows measurement of current or voltage changes across the membrane.	Sub-millisecond and subthreshold resolution, capable of identifying ionic conductances, action potentials and synaptic currents in identified individual cells. Electrophysiological, anatomical and molecular cell type identification possible. Can be combined with electrical or optogenetic perturbation to assess local and long-range connectivity. Combination with pharmacological agents can identify molecular mechanisms underlying normal firing.	*Ex vivo* brain slices is a highly isolated system, lacking interareal physiological brain dynamics. Can only focus on cellular mechanisms of single neurons.
***Ex vivo* multi-neuron patch-clamp**. Multiple neighbouring neurons in a brain slice are patched using the patch-clamp technique. Action potential in single neurons can be elicited by current injections. Simultaneous recording of the other neurons can detect correlated subthreshold synaptic potentials or currents indicative of a monosynaptic connection.	Unambiguous detection of monosynaptic connectivity and investigation of synaptic plasticity between identified neurons. Can be combined with optogenetic stimulation of afferent axons to map long-range input onto cell types.	Expensive and experimentally challenging technology. Sparse sampling of local population (up to 10 neurons).
***In vivo* patch-clamp**. Patch-clamp pipettes are lowered through a craniotomy into the brain of an awake head-fixed or anesthetized animal. Cells are targeted by monitoring the voltage fluctuation of the patch electrode without visual control (2-photon imaging guided possible). Same recording principle as *ex vivo* patch-clamp.	Detection of subthreshold membrane potential fluctuations and single action potentials of single cells in awake animals. Can be combined with optogenetic stimulation and cell type identification. Electrophysiological and anatomical characterisation possible.	Experimentally challenging approach with very limited throughput. Only single cell recording. Normally no visual control during the approach of neurons.
***In vivo* juxtacellular recording**. Similar approach as *in vivo* patch-clamp with the difference that the glass pipette is in a cell-attached configuration without rupturing the cell membrane. Spiking activity can be measured while electrical stimulation is used to introduce biotin through the membrane for post-hoc anatomical identification.	Detection of spiking activity of a single neuron *in vivo*. Recordings are more robust than wholecell patch-clamp recordings. Post-hoc anatomical identification of cell type possible.	No recording of subthreshold activity. Experimentally challenging with low throughput. No visual control during the approach of neurons.
**Extracellular electrical recordings**. Several single wires or tetrodes are inserted into the brain and are chronically implanted on the animal’s head. Electrodes can be held by *microdrives* that allow for dorso-ventral displacement. These electrodes provide recordings of the local field potential and spiking activity from neighbouring neurons.	Detection of single spikes from multiple neurons. Multiple electrodes can be inserted in different brain regions. LFP signals serve as a proxy for population activity. Implanted microdrives allow chronic recording in freely moving animals. Self-made wire electrodes are inexpensive.	Spike sorting cannot unequivocally guarantee that a single unit is the activity of a single neuron. No anatomical or molecular identification of recorded *units*. Low *unit* yield.
**High-density silicon probes. Commercially** manufactured probes containing single or multiple shanks are inserted into the brain of an head-fixed or anesthetized animal with a manipulator. Chronic implants are possible. LFP and spiking activity from many electrodes can be recorded.	Up to 384 simultaneous channels per probe allow a denser sampling of spiking neurons. Long shanks allow recording from multiple brain regions.	Single units cannot be anatomically or molecularly identified unless it is combined with optogenetic perturbations. Very expensive probes that can break, making chronic implants in freely moving animals rather challenging.
**Two-photon (2P) microscopy calcium imaging**. Identified individual cells and selected cellular population are imaged at superior resolution, thus revealing intricate details about neuronal activity within different cellular compartments.	Subcellular resolution, cell type- and pathway-specificity, activity within subregions of somatic, axonal, and dendritic compartments measurable. Proof-of-concept for compatibility with freely-moving experimental conditions and imaging of deep brain regions. Combination with optogenetics is possible.	Expensive and highly invasive technology, head-fixation often necessary, complex data analysis required.
**Microendoscopy**. Through an implanted graded refractory index (GRIN) lens, a headwearable microscope (miniscope) allows imaging of fluorescence changes of GECI within individual cells, thereby revealing parallel neuronal activity of up to hundreds of identified cells.	Cellular resolution, cell type- and pathway-specificity, activity of somatic and axonal compartments measurable. Deep brain regions accessible. Compatible with freely-moving experimental conditions. Combination with optogenetics is possible.	Invasive technology, large mechanical lesion by implantation of the lens, complex data analysis required.
**Photometry**. Implantable glass fibers coupled to excitation light sources and light-detecting sensors enable recording of fluorescence emitted by GECI or monoamines. Bulk imaging of calcium transients from many cells yields information about neuronal activity of entire cell populations or pathways.	Robust and inexpensive technology, cell-type- and pathway-specificity, activity of somatic and axonal compartments measurable. Deep brain regions accessible. Compatible with freely-moving experimental conditions. Combination with optogenetics is possible.	No cellular resolution, prone to movement artifacts.
**Optogenetics**. The activity of selected neurons is manipulated by expression of light-sensitive biomolecules (e.g. opsins) and light delivery via implantable (or external) light sources. A plethora of opsin variants allows precise temporal control of neuronal activity at the somatic or axonal level.	Robust and inexpensive technology, cell-type- and pathway-specificity, somatic and axonal compartments can be targeted. High temporal precision, allowing for insertion of physiologic activity patterns. Deep brain regions accessible. Reversible effect. Compatible with freely-moving experimental conditions.	Non-physiologic stimulation conditions, potential heating and off-target effects.
**Pharmaco-/chemogenetics**. The activity of selected neurons is manipulated by expression of artificial receptors, which are bound by systemically or locally injected ligands.	Robust and inexpensive technology, minimally invasive, cell type- and pathway-specificity, somatic and axonal compartments can be targeted. Reversible effect. Deep brain regions accessible. Compatible with freely-moving experimental conditions.	Non-physiologic stimulation conditions, potential unspecific ligand- and off-target effects, slow on and off kinetics.

**Table 2:** Molecular basis of modern circuit neuroscience

**Cre-driver lines and double floxed viral vectors** Cre-driver lines are transgenic mouse-lines, which express the enzyme Crerecombinase under the control of a tissue- or cell-type specific promoter. Crerecombinase removes or inverts DNA segments flanked by specific signal sequences, termed loxP-sites, depending on their orientation. Cre-driver lines can be combined with viral vectors carrying a “floxed gene” of interest in their genome. This floxed gene is placed in an inverted orientation and flanked by two pairs of non-homologous loxP sequences. The presence of Cre recombinase will allow for the irreversible change in orientation of the flanked DNA sequence enabling transcription of the floxed gene. Delivery of viral vectors is performed by stereotactic intracranial injection into the desired region. Utilization of the Cre/loxP, by combination of a Cre-driver line and floxed viral vectors, or the similar Flp/FRT system, enables cell-type specific expression of floxed genes such as calcium sensors, opsins (see below) or neuronal tracers. In the case of widely used adenoassociated viral (AAV) vectors, genes can be introduced, depending on the vector’s capsid and serotype, into neurons via the cell body or from the synaptic terminals, using the intracellular transport system to travel either antero- or retrogradely. Furthermore, viral infection can be either static (e.g. AAV vectors) or transsynaptic (e.g. Rabies vectors). Detection of introduced proteins is performed by immunohistochemistry *post mortem*. For further reference see (Haery et al., 2019; Navabpour et al., 2019).
**Genetically encoded calcium indicators** The increase of intracellular calcium concentration correlates with neuronal activity. The development of genetically encoded calcium indicators (GECIs), e.g. from the commonly used GCaMP-family, enabled dynamic visualization of calcium concentration changes (Lin and Schnitzer, 2016). In particular, the binding of calcium to the GCaMP molecule results in a conformational change, which then renders the protein excitable by photons of a specific wavelength thereby producing fluorescent emission. While the most widely used GCaMPs are excitable by blue light and emit green fluorescence, red-shifted variants have recently been developed and now allow for more flexibility in experimental designs (Helassa et al., 2016). Compared with direct electrical recordings however, calcium sensors reflect neuronal activity rather sluggishly, with relatively fast fluorescent rise times in the range of tens of milliseconds and slow decay times in the range of hundreds of milliseconds. GCaMPs are constantly improved for better signal-to-noise ratio and faster kinetics, and will potentially resolve high firing rates with single action potential resolution in the future (Mollinedo-Gajate et al., 2019).
**Opsins** Opsins are light-sensitive membrane channels/receptors, which change their spatial conformation to increase their opening probability for specific ions when stimulated by a defined light wavelength. They can be either conductive for cations (e.g. Channelrhodopsin,ChR2) or negatively charged ions (e.g. Halorhodopsin,NpHR)). Depending on the ion flux through the cell membrane, the intracellular electrical and chemical balance is changed, resulting in de- or hyperpolarization, and subsequent enhanced or decreased spiking activity, respectively. Systematic screening of natural light-sensitive molecules, combined with targeted genetic modifications, has created an entire toolbox of various opsins with different light sensitivity and kinetics. These encompass ultrafast activation patterns in the millisecond range (Mager et al., 2018) up to long-lasting channel opening times over minutes in so-called step-function opsins (Berndt et al., 2009). Opsins can be sensitive to light with short wavelengths in the blue range (e.g. ChR2), which transmits high photon energy but is quickly absorbed and scattered in biological tissues. Alternatively, other opsin variants (e.g. ReaChR, Chrimson, ChRmine) are sensitive to lower energy red light, with higher tissue penetrability. Recent developments allow reliable activation and inhibition of the same cells with balanced stoichiometry (Vierock et al., 2021). For further reference see (Fenno et al., 2011; Tye and Deisseroth, 2012).

## Abbreviations

AAV: Adeno-associated virus
AP: Action potential
CB: Cerebellum
ChR: Channelrhodopsin
CMOS: Complementary metal-oxide-semiconductor
DBS: Deep brain stimulation
EPN: Entopeduncular nucleus
EPSP/IPSP: Excitatory/inhibitory postsynaptic potential
GECI: Genetically encoded calcium indicator
GPe/i: Globus pallidus externus/internus
HFS: High frequency stimulation
L-DOPA: Levodopa
LFP: Local field potential
LID: Levodopa-induced dyskinesia
M1: Primary motor cortex
MLR: Mesencephalic locomotor region
PAG: Periaqueductal grey
PD: Parkinson’s disease
Pitx2: Paired-like homeodomain 2
PV: Parvalbumin
SNr/c: Substantia nigra pars reticularis/pars compacta
SPN: Striatal projection neuron (medium spiny neuron)
dSPN: Direct pathway striatal projection neuron
iSPN: Indirect pathway striatal projection neuron
SST: Somatostatin
STN: Subthalamic nucleus
STR: Striatum
6-OHDA: 6-Hydroxydopamine
TH: Thalamus
ZI: Zona incerta
